# Notes for Contributors

**DOI:** 10.1155/S1463924679000389

**Published:** 1979


enables the operator to choose from a variety of operational modes and to interact with the instrument in order to achieve optimum performance.

des donnees. La flexibilité du "software" permet a l'opérateur de choisir parmi un grand nombre de modes opérationnels d'une facon interactive avec l'instrument à fin d'obtenir une performance optimale.

matische Probenbehändigung, und Daten-Analyse ermöglicht. Die Flexibilität der Steuerung durch Software ermöglicht der Bedienungsperson eine Reihe von Betrieb-sarten anzuwahlen und auf das Instrument Einfluss zu nehmen, um eine optimale Leistung zu erzielen.

**Page 140**

**An evaluation of the Kem-O-Mat programmable discrete analyser**

Geoffrey C. Seymour

The Kem-O-Mat is a single channel discrete analyser which is operated under control from a programmable calculator. The programmes can either be supplied by the instrument manufacturer or developed by the operator thereby facilitating the use of the laboratory's own methods on the instrument. The volumes of sample and reagents required for the chemistries are small, thus affording a net saving in running costs. The instrument has been well received by the laboratory staff for several reasons; mainly, simplicity of operation, ease of maintenance, versatility, fast through-put and improved performance with regard to precision in the author's laboratory.

**Evaluation de l'analyseur programmable discontinu Kem-O-Mat**

Le Kem-O-Mat est un analyseur discontinu à un canal qui fonctionne sous le contrôle d'un calculateur programmable. Les programmes peuvent être livrés soit par le producteur soit developpés par l'utilisateur pour meilleur adaption des méthodes propres a son laboratoire. Les volumes des échantillons et des reactifs requis sont bas, ainsi ils reduisent les frais courants. L'inst-rument a été bien reçu par le personnel du laboratoire pour les raisons suivantes: simple opération, peu maintien, sa faculté d'adapt-ation , son grand débit et pour les resultats plus precis obtenus dans le laboratoire de l'auteur.

**Eine Beurteilung des programmierbaren, diskontinuierlichen Kem-O-Mat Analysators**

Der Kem-O-Mat ist eindiskoantinuierlicher Einkanalanalysator der von einem programmierbaren Rechner gesteuert wird. Die Programme konnen entweder vom Instrumentenhersteller geliefert werden oder aber können durch die Bedienungsperson entwickelt werden, womit die Benutzung der im Laboratorium verwendeten Methoden auf dem Instrument erleichtert wird. Die für die Chemie benötigten Volumina vol Probe und Reagenzien sind klein, was eine Senkung der Betriebskosten erlaubt. Das Instrument wurde vom Laboratoriumspersonal gut akzeptiert aus den folgenden Gründen: Einfachheit der Arbeitsweise, leichter Unterhalt, Vielseitig-keit, hoher Durchsatz und verbesserte Leistungen. im Bezug auf Präzision im Laboratorium des Autors.

### Notes for Contributors

### Presentation of manuscripts

Manuscripts should be typed (double-spaced) on one side of the paper only and with generous margins. The title should be brief and informative avoiding the word "new" and its synonyms. The full list of authors with their affiliations and full address(es) should appear on the title page. On a separate sheet an abstract of no more than 150 words is required. This should succinctly describe the scope of the contribution and highlight significant findings or innovations. It should be written in a style which can easily be translated into French and German.

*The Concise Oxford Dictionary* and Fowler's *Modern English Usage* (both published by Oxford University Press) should be used as the standard for spelling and grammar. Abbreviations should be limited to those generally recognised, or where a frequently occuring term is abbreviated it should, in the first instance, be explained thus "flow injection analysis (FIA) ..." and the abbreviation used thereafter. Abbreviations, for standard measures and units should follow SI recommendations. There are various publications giving guidance on the use of SI units.

References should be indicated in the text by numerals following the author's name, i.e. Skeggs [6]. On a separate sheet of paper, list all references in numerical order thus:

[6] Skeggs, L.T., *American Journal of Clinical Pathology,* 1959, **28**, 311.

Note that journal titles are given in full. Where there is more than one author, the form Foreman et al. should be used in the text but all authors should be named in the list of references. When reference is made to a chapter in a book the reference should take the following form:

[7] Malmstadt, H.V. in "Topics in Automatic Chemistry" Ed. Stockwell P.B. and Foreman J.K. 1978 Horwood, Chichester, pp. 68-70.

Only work which has been published or has been accepted for publication should be cited. Avoid the citation of documents which are subject to restricted circulation, patent literature, unpublished work and personal communications. The latter can be mentioned in the text in parenthesis.

To illustrate a paper line diagrams are preferred to photographs. Photographs should only be used when they significantly add to the discussion. Diagrams, charts and graphs should be carefully drawn in black ink on stout card or heavy quality tracing paper. Most illustrations are reduced for publication; to allow for this originals should be between 16 and 36 cm wide (the depth must not exceed 50 cm). The lettering of diagrams should be sufficiently clear to withstand reduction. Except in the case of proper names, all lettering should be in lower case print. If photographs are used they must be supplied in the form of clear, unmounted, glossy, black and white prints. "Instant" photographs are not normally acceptable. All illustrations must be identified on the reverse showing the figure number and the author's name.

Each illustration should have a fully explanitory caption. Captions should be typed together on a separate sheet of paper; they must *not* be an inseparable part of the illustration.

